# Influence of skin pigmentation on the accuracy and data quality of photoplethysmographic heart rate measurement during exercise

**DOI:** 10.1007/s00421-025-05977-x

**Published:** 2025-09-18

**Authors:** Anne M. Mulholland, Hayley V. MacDonald, Elroy J. Aguiar, Jonathan E. Wingo

**Affiliations:** 1 Department of Exercise Science, Mercer University, Macon, GA USA; 2 Department of Kinesiology, The University of Alabama, Tuscaloosa, AL USA

**Keywords:** Skin color, Wearable device, Wearable technology, Physiological monitoring

## Abstract

**Introduction:**

Wearable devices often use photoplethysmography to estimate heart rate (HR) by measuring light reflected from the cutaneous vascular bed. Notably, higher melanin content of the epidermis may reduce the amount of light transmitted through the skin. Previous studies examining the impact of skin tone on photoplethysmographic HR measurement accuracy have produced mixed results; however, none measured epidermal melanin content.

**Purpose:**

To determine whether objectively measured skin pigmentation influences the accuracy of photoplethysmographic HR measurement during rest, exercise, and recovery.

**Methods:**

Skin pigmentation was quantified from colorimeter measures using Individual Typology Angle (ITA°), a strong correlate of epidermal melanin, in 28 healthy adults (White, *n* = 16; Black, *n* = 10; Middle Eastern, *n* = 1; Persian, *n* = 1). Chest-worn HR (criterion; Polar H10) and photoplethysmographic HR from three devices (Apple Watch Series 8, Garmin vivosmart 5, SlateSafety BAND V2) were recorded continuously during rest, cycling, and recovery; HR data were averaged into 30-s epochs for analysis. A linear mixed-effects model determined whether ITA° influenced mean absolute error of HR (MAE_HR_) for each device.

**Results:**

ITA° predicted MAE_HR_ for SlateSafety (β =  − 0.011, *P* = 0.001), but not for Apple (*P* = 0.62) or Garmin (*P* = 0.29). Missing data were disproportionately attributed to participants with dark skin (ITA° < 10°; 36%) for Apple (50%) and SlateSafety (85%) devices; outliers were disproportionately observed among participants with dark skin for all devices (56%–62%).

**Conclusion:**

Data quality was negatively impacted by darker skin pigmentation for all tested devices, but skin pigmentation (ITA°) only increased photoplethysmographic HR measurement error to a small degree (~ 1 bpm) for the SlateSafety device.

**Supplementary Information:**

The online version contains supplementary material available at 10.1007/s00421-025-05977-x.

## Introduction

Wearable devices are becoming increasingly popular for non-invasive monitoring of physiological responses to physical activity and exercise, with global sales expected to reach $186 billion USD by 2030 (Doherty et al. 2024). Many of these devices estimate heart rate (HR) using photoplethysmography, which employs a light-emitting diode (LED) and a photodetector that senses the variation of the light reflectance at the epidermis due to the pulsatile nature of cutaneous arterial blood flow (Biswas et al. 2019). Green LED (wavelengths of ~ 525 nm) is most often used for wearable devices but melanin reduces absorption of light at that wavelength (Zonios et al. 2001). As such, high epidermal melanin content may pose a challenge for accurate optical HR measurement. A systematic review by Koerber et al. (2023) documented conflicting results regarding the influence of skin pigmentation on the accuracy of optical HR measurement by commercially available devices (e.g., Garmin, Fitbit, Apple watches). Of the ten studies included, four studies reported no differences across skin tones, four studies reported that darker skin was associated with less accurate optical HR measurement compared to lighter skin, and the remaining two studies reported mixed results. All the studies included by Koerber et al. (2023) shared one substantial methodological limitation: the use of categorical ratings to subjectively characterize skin pigmentation. Subjective rating scales of skin color have shown poor discrimination by clinicians between adjacent categories (Pershing et al. 2008) and poor intra-rater reliability (Hannon and DeFina 2020). Indeed, even the Fitzpatrick Skin Type (Fitzpatrick 1988), one of the most popular and widely used rating scales for skin color description, has demonstrated highly variable results for measured skin pigmentation within a designated category, especially for more darkly pigmented skin (Branigan et al. 2013).

A viable alternative to subjective rating scales is colorimetry, an objective method that provides a quantitative measure of skin pigmentation as Individual Typology Angle (ITA°) (Pershing et al. 2008; Ware et al. 2020; Colvonen et al. 2020). Colorimeters are used in dermatological settings to quantify skin pigmentation for a variety of medical and cosmetic purposes (Ly et al. 2020). Beyond the initial cost, which can be over $20,000, the measurement itself is simple, quick, non-invasive, and provides an index of skin pigmentation that is free from rater bias when measurements are properly standardized.

Despite the utility and objectivity of colorimetry, no studies have investigated the influence of measured skin pigmentation on the accuracy of photoplethysmographic HR. As such, the purpose of this study was to quantify the extent to which photoplethysmographic HR differs from a criterion measure of HR across a wide range of objectively measured skin pigmentation during rest, exercise, and recovery. Darker skin pigmentation was hypothesized to result in greater error of photoplethysmographic HR measurement compared to lighter skin pigmentation.

## Methods

### Experimental design

The present study is a criterion validity study. All procedures were approved by The University of Alabama’s institutional review board, and all participants provided written informed consent prior to commencing the study.

### Participants

We performed an a priori power analysis in G*Power 3.1.9.6 (Faul et al. 2007). To detect a 5-bpm difference in HR between the criterion and photoplethysmographic devices for the proposed study design, an effect size equal to *d* = 0.18 was estimated using means and standard deviations from our pilot data (*n* = 12) (Mulholland et al. 2024). The magnitude of this small effect size is in agreement with those reported by Sañudo et al. (2019), which ranged from 0.05 to 0.27. We first used a repeated measures model, with two groups (representing two devices—criterion and test) and six measurements (representing six exercise intensities), assuming a high correlation among repeated measures (*r* = 0.9), an alpha level of 0.05, and a power level of 0.8. An effect size of 0.2 produced an estimated sample size of 30 participants, and similarly, power analyses for linear multiple regression (alpha level = 0.05, power = 0.8, predictors = 2) would require 52 observations.

Healthy men and women aged 18–59 y who were free of cardiovascular, metabolic, and renal disease and who did not need medical clearance according to the American College of Sports Medicine pre-participation screening algorithm (American College of Sports Medicine 2021) were recruited to participate. Exclusion criteria included the presence of hypertension [resting systolic blood pressure (BP) ≥ 130 mm Hg or diastolic BP ≥ 80 mm Hg, currently taking antihypertensive medication, or having been told by a medical provider they have high BP on ≥ 2 separate occasions]; tachycardia (resting HR > 100 bpm); current cigarette smoking or nicotine use, or quit < 6 months prior; the use of medication that alters HR, skin blood flow, sweat rate, or metabolic responses to exercise; and tattoos or scars at any wearable device measurement site (wrist and upper arm). To ensure adequate sample distribution across the spectrum of skin tones, we set an a priori target that at least 40% of included participants were self-reported persons of color and had an average ITA° of 10° or less (dark skin) at the device placement sites. ITA° is highly correlated (*R*^*2*^ = 0.96) with total melanin content of the epidermis (Del Bino et al. 2015). ITA° was calculated from colorimeter measurements using Eq. (1),

1 $$ITA^\circ =\mathit{arctan}\left(\frac{{L}^{*}-50}{{b}^{*}}\right)\times \frac{180}{\pi }$$

where L^*^ is the luminance value and b^*^ is the yellow-blue component (Ly et al. 2020).

Participants were instructed to arrive well rested, hydrated, and having refrained from ingesting non-prescription drugs on the day of testing. In addition, participants refrained from consuming alcohol and caffeine, from participating in strenuous exercise for ≥ 24 h prior to testing, and from using artificial tanning lotion or similar products for ≥ 1 week prior to study participation.

### Experimental procedures

Upon arrival at the laboratory, participants completed a self-reported physical activity history, medical history, and a 24-h history questionnaire. Women self-reported the first day of previous menses and were tested during the follicular phase of the menstrual cycle; menstrual cycle phase was not expected to influence study outcomes (Stone et al. 2021).

After resting quietly for 5 min in a seated position, resting BP was measured according to the procedures outlined by the American Heart Association (Whelton et al. 2018). Following BP measurement, a colorimeter (observer = 2°, illuminant = C; Chromameter CR-400, Konica Minolta, Ramsey, NJ) was used to measure skin pigmentation at the volar (inner) upper arm for standardization (Pershing et al. 2008) and at each photoplethysmographic device measurement site—the posterior wrist and lateral upper arm—following the procedures outlined in Ly et al. (2020). Prior to measurement, each site was cleaned with an alcohol swab, and excessive body hair was removed if necessary. Colorimeter measurements were taken in triplicate at each site by the same researcher for all participants, with the colorimeter held perpendicular to the skin, and the average value for each term was used for all calculations and analyses.

Next, participants provided a urine void that was used to measure urine specific gravity (USG) with a refractometer (PAL-10S, Atago, Tokyo, Japan); USG ≤ 1.020 was considered adequately hydrated (Sawka et al. 2007). After the urine sample was provided, nude body mass was measured using a digital scale (BWB-800, Tanita Corporation, Tokyo, Japan). Standard clothing (tank top and cycling shorts) was provided for all participants to wear during exercise. Height was measured using a stadiometer (model 213, seca, Hamburg, Germany) and body composition was estimated using the 7-site sum of skinfolds (Lange skinfold caliper, Beta Technology, Inc., Santa Cruz, CA) (Jackson and Pollock 1985).

Participants were then outfitted with a chest-strap HR monitor (H10, Polar, Finland) for the criterion measurement of HR (Gilgen-Ammann et al. 2019). Next, participants were instrumented according to manufacturers’ instructions with the wearable devices to be tested: BAND V2 (SlateSafety, Atlanta, GA) on the lateral upper non-dominant arm, the vivosmart 5 (Garmin, Kansas City, MO) on one wrist, and the Apple Watch Series 8 (Apple Inc., Cupertino, CA) on the other wrist; device placements between the dominant and non-dominant wrists were counterbalanced and the counterbalanced placement orders were randomly assigned. These particular watches were chosen because they were, at the beginning of the study in 2023, the newest models of popular brands for fitness trackers at different price points (Apple Watch Series 8, $399; Garmin vivosmart 5, $149 at the time of purchase in 2023). The SlateSafety BAND V2, also the newest model available at the onset of data collection, was included because of its marketed use (occupational monitoring), where potential bias in its HR measurement is important to identify from a health and safety standpoint.

Participants then entered a room maintained at an ambient temperature of 22.4 ± 0.1 °C and a relative humidity of 41% ± 4%. The participant was seated for 5 min while baseline measures for criterion HR and data from all wearable devices were recorded. Next, participants mounted the cycle ergometer (Excalibur Sport, Lode, Netherlands) and completed a graded protocol. Cycling was chosen as the mode of exercise to minimize the influence of movement artifact on HR measurement. The protocol consisted of four 10-min stages, for a total of 40 min of continuous exercise. The four stages were completed at 50%, 60%, 70%, and 80% of age-predicted maximal HR (HR_max_) (Tanaka et al. 2001); these stages corresponded to very light (< 57% HR_max_), light (57%–63% HR_max_), moderate (64%–76% HR_max_), and vigorous (> 76% HR_max_) intensity exercise, respectively (American College of Sports Medicine 2021). The purpose of the graded protocol was to be able to compare device performance across a range of exercise intensities and absolute HR values.

During exercise, criterion HR and data from all wearable devices were recorded continuously. Work rate was adjusted so that target HR was achieved within 2 min of beginning each stage and further adjusted to maintain target HR throughout each stage. Oxygen uptake (V̇O_2_) was measured for 2 min starting at the 5th min of each stage using indirect calorimetry (TrueOne 2400, PARVOMedics, Salt Lake City, UT), then converted to metabolic equivalents (METs; 1 MET = 3.5 mL·kg^−1^·min^−1^). During the last min of each stage, rating of perceived exertion (RPE) was collected (Borg 1982). After 40 min, participants stopped cycling and moved to a chair where they remained seated for 10 min to measure responses during post-exercise recovery. Following the recovery period, participants were de-instrumented.

### Device setup and data cleaning

Criterion HR from the Polar H10 monitor was recorded using the Polar Beat app (version 3.5.6) and each session was exported from the diary on the Polar Flow website. Polar records HR once per second. For the Apple (watchOS version 9.6) and Garmin (software versions 3.02, 3.13, 3.14, 3.22) watches, an indoor cycling workout was recorded for the duration of the protocol, started prior to resting measurements and ended after the 10-min recovery. Apple HR data were synced and exported from the iPhone Health App (iOS version 16.5.1). Apple records HR approximately once every 5 s during a workout. The Garmin workout was downloaded from the device using the Garmin Connect app (version 4.69.1.5) and then exported as a.tcx file from the Garmin Connect website. An open source .exe script was used to extract time and HR data for each workout (available from http://www.wartnaby.org/running/file_converters/index.html). Garmin records HR every 2 to 15 s during a workout. SlateSafety data were downloaded from their online BioTrac platform after each session; the SlateSafety BAND V2 (software versions 1.6.1, 1.6.2, 1.6.3) records HR once every 10 s. Throughout the study, Garmin (3 updates) and SlateSafety (2 updates) pushed firmware/software updates to their respective devices/platforms with no option to opt out. Updates were not expected to impact study results.

Prior to analysis, raw data files were checked for HR values < 50 bpm or > 200 bpm. Any instances of likely erroneous measurements were manually inspected and removed if appropriate. All HR data were then averaged into 30-s epochs to account for differences in sampling frequency. Missing data rates were counted as the number of 30-s epochs that were not calculated because there were no data available. Each device was synced with a wireless internet or cellular network-enabled iPhone immediately prior to each data collection session. Time stamps were exported with HR data and pooled in the same 30-s epochs as HR data. Summarized data from all devices were then matched using time stamps. Outliers were identified as 3.29 × SD of the mean error of HR (Field 2024) and were removed prior to analysis.

### Data analysis

All statistical analyses were completed using R Statistical Software version 4.4.1 (R Core Team 2024). Descriptive statistics (mean ± SD) were generated for all indicated outcome measures. HR data were evaluated at six intensities: rest, very light, light, moderate, vigorous, and recovery. The magnitude of error at each intensity was characterized as mean absolute error of HR measurement (MAE_HR_), the absolute value of the difference in mean criterion HR and mean device HR for a 30-s epoch. Paired samples *t* tests were used to confirm target HR was achieved by the criterion HR measure during exercise. Overall device performance—HR measurement by each device versus criterion HR measurement—was described using a repeated measures correlation coefficient (*r*_*rm*_) (Bakdash and Marusich 2017) and Bland–Altman agreement analysis for repeated measures (Bland and Altman 2007) with modified Bland–Altman plots (Krouwer 2008). Proportional bias was calculated as the trend (Pearson’s *r*) between the difference in HR measurements and criterion HR from the modified Bland–Altman plots (Krouwer 2008). For this study, 95% limits of agreement (LoA; equal to bias ± 1.96 × SD) ≤ 5 bpm was considered “excellent,” > 5 to ≤ 10 bpm was “good,” and > 10 bpm was “unacceptable” agreement; these thresholds were chosen because they represent 5% error at a HR of 100 bpm and 200 bpm, respectively. In addition, the American National Standard Institute (2002) requires experimental devices intended for use in a medical setting to be within ± 10% or ± 5 bpm (whichever is greater) of a criterion measure for HR, and ± 5% is a commonly used threshold for wearable devices measuring step-related metrics that has also been applied to HR measurement (Fokkema et al. 2017; Shcherbina et al. 2017).

A linear mixed-effects model (Bates et al. 2015) was used to determine whether skin pigmentation (as ITA°) predicts MAE_HR_; a multilevel modeling approach was employed to account for repeated measures within participants. MAE_HR_ for each device was evaluated in a separate model. The intraclass correlation coefficient (ICC) was calculated to describe the percentage of variance existing at the participant level (i.e., between-person variance). Model building was completed using a forward entry method to evaluate whether 1) ITA° or 2) criterion HR explained additional variance in MAE_HR_. A likelihood-ratio test was performed to determine whether each predictor should be kept in the model. The marginal *R*^2^ value was calculated to describe the proportion of variance explained by the fixed effects (coefficients) in each model. Normality was assessed by visual inspection of Q-Q plots, and heteroscedasticity was assessed by visual inspection of residuals vs. fitted plots. Variance inflation factor (VIF) statistics were calculated to test for multicollinearity, with VIF < 5 considered acceptable. Squared semi-partial correlations (*sr*^2^) were calculated for significant predictor variables to describe the percentage of model variance uniquely explained by each predictor. To compare physiological responses and MAE_HR_ (within each device) across intensities, a one-way analysis of variance was used; in the event of a significant omnibus test, pairwise comparisons with a Bonferroni α correction were performed. If sphericity was violated, the Greenhouse–Geisser correction was applied. All statistical tests used an α level of 0.05.

## Results

Thirty individuals were consented and twenty-eight (54% female) participants completed all study procedures (mean ± SD, age = 25 ± 5 y; body mass = 74.5 ± 15.0 kg; height = 1.72 ± 0.08 m; body fat = 21% ± 8%; age-predicted HR_max_ = 191 ± 4 bpm). Participants had normal BP (systolic BP = 116 ± 6 mm Hg; diastolic BP = 71 ± 5 mm Hg) and were adequately hydrated (USG = 1.013 ± 0.011) prior to exercise.

Mean ITA° at the volar arm was 31.4° ± 35.5° (range, − 44.9° to + 69.1°) and at the device measurement sites was 11.9° ± 39.2° (− 63.2° to + 63.4°); ten (36%) participants had an average ITA° at all device placement sites of < 10° (− 36.8° ± 16.3°). Mean ITA° at each device placement is reported in Table 1.

**Table 1:** Descriptive characteristics for participant race, ethnicity, and skin pigmentation

Race^a^	*n* (%)^b^	
Black	10 (36%)	
White	16 (57%)	
Middle Eastern	1 (4%)	
Persian	1 (4%)	
Ethnicity^a^		
Hispanic	1 (4%)	
Non-Hispanic	27 (96%)	
Person of color^a^	13 (46%)	
Skin pigmentation (ITA°)^c^		
Device	Mean ± SD	Range
Apple Watch Series 8	13.0 ± 38.8	− 57.8 to + 59.4
Garmin vivosmart 5	11.7 ± 39.1	− 63.2 to + 58.7
SlateSafety BAND V2	11.1 ± 41.1	− 55.8 to + 63.4

^a^Self-reported

^b^Percentages may not sum to 100% due to rounding

^c^Measured using a colorimeter and calculated as Individual Typology Angle (ITA°). ITA° is reported separately for each device at the wrist (Apple and Garmin) and upper arm (SlateSafety)

### Data quality

Some HR data were unavailable from the recorded sessions for the Apple Watch (7%), the Garmin vivosmart (20%), and the SlateSafety BAND (20%). No Polar H10 data were missing. Most of the missing Apple and Garmin data were lost due to data recording errors (i.e., the workout did not record) and Garmin data from two participants were unable to be downloaded from the device after recording (accounting for ~ 8% of the lost Garmin data). No apparent cause for data loss could be identified for the remaining Apple (2%), Garmin (2%), or all the missing SlateSafety data, and missing data were irrecoverable. Missing data not attributable to data recording errors were disproportionately from participants with dark skin (ITA° < 10°) for the Apple (50%) and SlateSafety (85%) devices, but not Garmin (33%). There were a total of 31 (1%) outliers in the Apple data, 45 (2%) in the Garmin data, and 69 (3%) in the SlateSafety data. Outliers were disproportionately represented in data from participants with dark skin for all devices: Apple (61%), Garmin (56%), SlateSafety (62%). Additional details about data quality are provided in Online Resource 1.

### Responses during rest, exercise, and recovery

Prior to starting exercise, criterion HR at rest was 69 ± 12 bpm. Based on the criterion HR measure, target HR was achieved for very light and light intensity exercise (both *P* > 0.05; Table 2); target HR was on average 3 bpm lower than criterion HR for moderate intensity (*P* < 0.001; Table 2) and 2 bpm lower than criterion HR for vigorous intensity (*P* = 0.04; Table 2). As expected, increases in exercise intensity required increases in external work rate and resulted in greater V̇O_2_ and higher METs over time (Table 2). After exercise, recovery HR by the criterion device was 100 ± 19 bpm.

**Table 2:** Mean ± SD target heart rate (HR), criterion HR, external work rate, and metabolic responses during exercise for all participants

	Very light	Light	Moderate	Vigorous
Target HR (bpm)	95 ± 2	114 ± 2	133 ± 3	153 ± 3
Criterion HR (bpm)	98 ± 10*^,†,‡^	113 ± 8^†,‡^	130 ± 8^‡,§^	151 ± 9^§^
$\dot{W}$ (W)	40 ± 17*^,†,‡^	66 ± 27^†,‡^	88 ± 35^‡^	108 ± 38
${\dot{\mathrm{V}}\mathrm{O}}_{{2}}$ (L·min^−1^)	0.9 ± 0.3*^,†,‡^	1.2 ± 0.4^†,‡^	1.4 ± 0.5^§^	1.8 ± 0.5
METs	3.5 ± 0.9*^,†,‡^	4.6 ± 1.3^†,‡^	5.6 ± 1.7^§^	6.8 ± 1.8
RPE	10 ± 2*^,†,‡^	12 ± 1^†,‡^	14 ± 1^§^	16 ± 2

$\dot{W}$, external work rate; ${\dot{\mathrm{V}}\mathrm{O}}_{{2}}$, oxygen uptake; METs, metabolic equivalents; RPE, rating of perceived exertion

*P < 0.05 compared to light intensity

^†^*P* < 0.05 compared to moderate intensity

^‡^*P* < 0.05 compared to vigorous intensity

^§^*P* < 0.05 compared to target HR at the same intensity

### Device accuracy

**Fig. 1:**
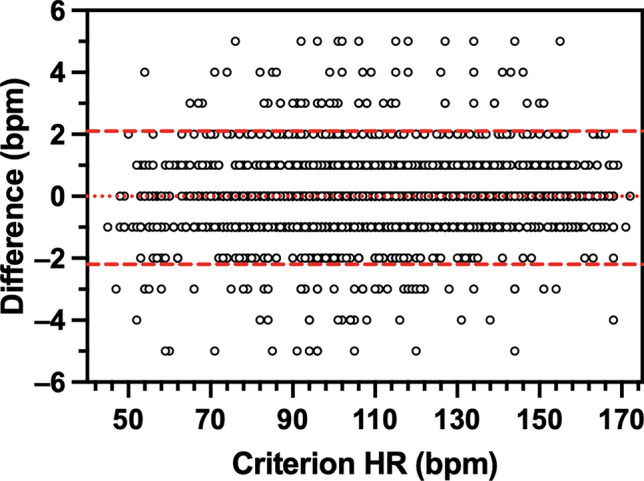
Modified Bland–Altman plot comparing heart rate (HR) measurement from the Polar H10 (criterion device) and the Apple Watch Series 8. The y-axis is the difference (Apple–Polar) in HR measures. The dotted line denotes overall bias and the dashed lines represent 95% limits of agreement

**Fig. 2:**
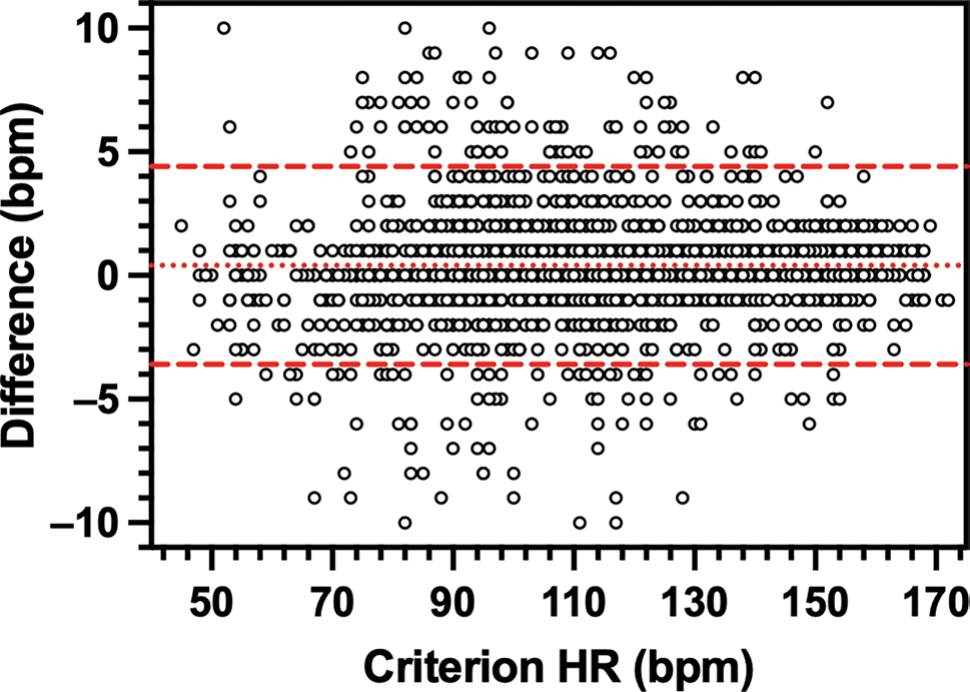
Modified Bland–Altman plot comparing heart rate (HR) measurement from the Polar H10 (criterion device) and the Garmin vivosport 5. The y-axis is the difference (Garmin–Polar) in HR measures. The dotted line denotes overall bias and the dashed lines represent 95% limits of agreement

**Fig. 3:**
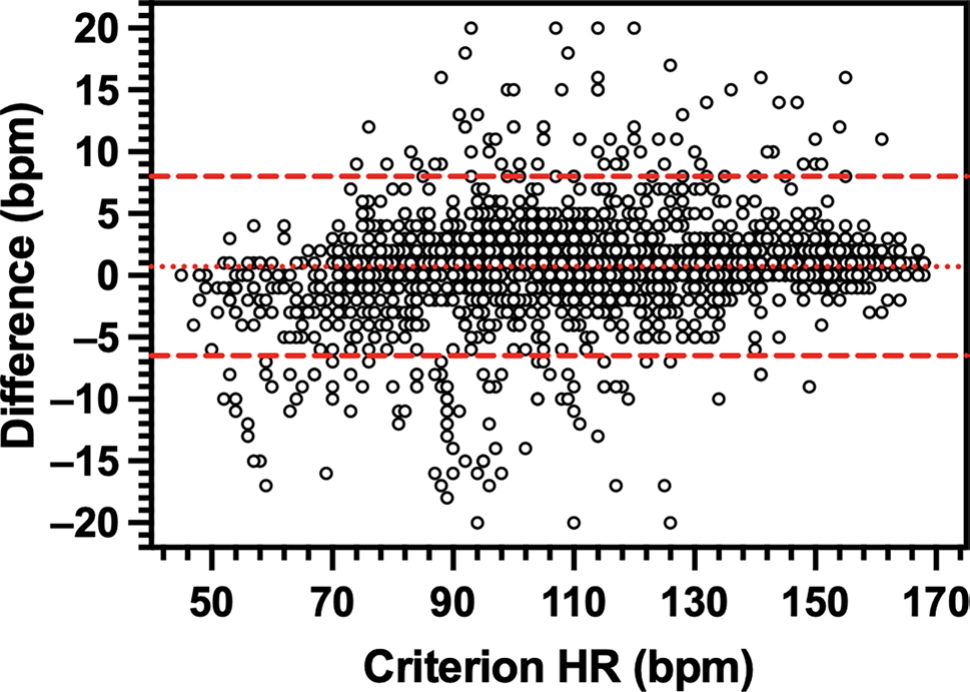
Modified Bland–Altman plot comparing heart rate (HR) measurement from the Polar H10 (criterion device) and the SlateSafety BAND V2. The y-axis is the difference (SlateSafety–Polar) in HR measures. The dotted line denotes overall bias and the dashed lines represent 95% limits of agreement

**Fig. 4:**
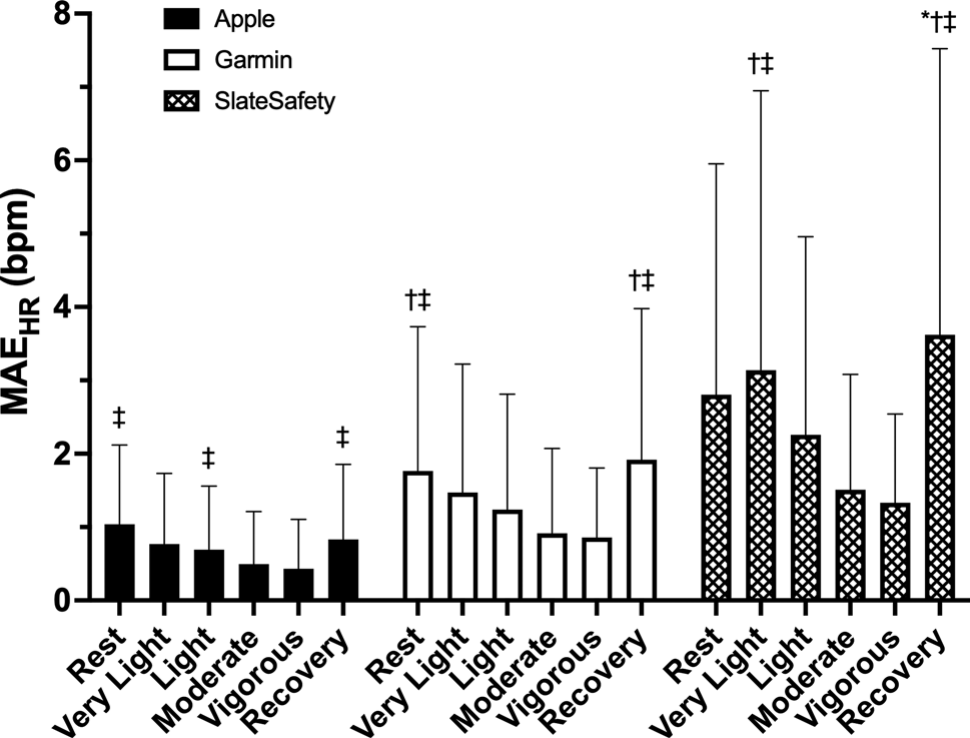
Mean ± SD mean absolute error of heart rate measurement (MAE_HR_) between the criterion device (Polar H10) and each photoplethysmographic device during rest, exercise (very light, light, moderate, and vigorous intensities), and recovery. *P < 0.05 compared to light intensity within the same device; ^†^*P* < 0.05 compared to moderate intensity within the same device; ^‡^*P* < 0.05 compared to vigorous intensity within the same device

HR measured by each wearable device was very strongly correlated with criterion HR (Apple, *r*_*rm*_ = 1.00, *P* < 0.001; Garmin, *r*_*rm*_ = 1.00, *P* < 0.001; SlateSafety, *r*_*rm*_ = 0.99, *P* < 0.001). Bland–Altman agreement analysis revealed that on average, the Apple Watch demonstrated no directional bias to the criterion measure while the Garmin vivosmart and the SlateSafety BAND overestimated HR, albeit by less than 1 bpm (Table 3). Based on the a priori thresholds established for 95% LoA, excellent agreement was observed for the Apple and Garmin devices (Table 3, Figs. 1 and 2), and good agreement was observed for the SlateSafety BAND (Table 3, Fig. 3). There was a small-to-moderate positive trend for all three tested devices, indicating proportional bias whereby HR was underestimated to a greater degree at lower HR values (Table 3). Average MAE_HR_ was higher at lower intensities but remained under 5 bpm for all devices at all intensities (Fig. 4). Mean absolute percent error data for all devices are provided in Online Resource 2.

**Table 3:** Bland–Altman agreement analysis results for heart rate (bpm) measurement from photoplethysmographic devices

			95% LoA		
Device	Bias	SD	Lower	Upper	Trend (*r*)
Apple Watch Series 8	0.0	1.1	− 2.2	2.1	0.10^*^
Garmin vivosmart 5	0.4	2.0	− 3.6	4.4	0.04^*^
SlateSafety BAND V2	0.7	3.7	− 6.5	8.0	0.18^*^

LoA, limits of agreement

^*^P < 0.05 for proportional bias (determined from modified Bland–Altman plot)

### Influence of skin pigmentation on device accuracy

For each wearable device, a linear mixed-effects model was used to determine significant predictors of MAE_HR_, with final models shown in Table 4. Full model-building results for each device are provided in Online Resource 3. When accounting for repeated measures within participants, 3%–6% of the total variance (marginal R^2^) in MAE_HR_ was explained by the included predictor variables (Table 4). Criterion HR was a significant predictor of MAE_HR_ for all devices, with an increase in HR associated with a small decrease in MAE_HR_; for instance, increasing HR by 100 bpm decreased MAE_HR_ by 2 bpm or less. For the SlateSafety BAND V2, ITA° was also a significant predictor of MAE_HR_ (Table 4). However, the magnitude of this relationship was small; across the wide range of skin pigmentations tested (ITA° range: − 56° to + 63°), MAE_HR_ was only ~ 1 bpm greater for individuals with the darkest skin compared to the lightest skin.

**Table 4:** Final models for prediction of mean absolute error in heart rate measurement (MAE_HR_) by each photoplethysmographic device

			Coefficients^a^			*sr*^*2*^	
Device	ICC	Constant	ITA°	Criterion HR (bpm)	Marginal *R*^*2*^	ITA°	HR
Apple Watch Series 8	0.074	1.433	–	− 0.006	0.034	–	0.03
Garmin vivosmart 5	0.074	2.779	–	− 0.013	0.040	–	0.04
SlateSafety BAND V2	0.049	5.160	− 0.011	− 0.022	0.058	0.02	0.03

HR, heart rate; ICC, intraclass correlation coefficient; ITA°, Individual Typology Angle; *sr*^*2*^, squared semi-partial correlation coefficient

^a^only significant predictors of MAE_HR_ are included

## Discussion

The purpose of this study was to test the hypothesis that darker skin pigmentation (lower ITA°) would reduce the accuracy of HR measured by photoplethysmographic devices during rest, exercise, and recovery because of the higher concentration of melanin and accompanying impairments of light transmission through the epidermis. In accordance with our hypothesis, ITA° was a significant predictor of MAE_HR_ for the SlateSafety BAND V2, although the overall magnitude of the effect was small (~ 1 bpm). Contrary to our hypothesis, ITA° was not a significant predictor of MAE_HR_ for the Apple or Garmin watches. However, there was a disproportionately higher rate of unexplained missing data for the Apple and SlateSafety devices and a higher incidence of outliers from all three devices for participants with dark skin (ITA° < 10°). Narrower LoA for the Apple and Garmin devices revealed better overall agreement with criterion HR compared to the SlateSafety BAND, although all devices produced acceptable agreement (when defined as LoA ± 10 bpm or better) with criterion HR.

The present findings for the Apple and Garmin watches are consistent with four studies that also found no effect of skin color (measured subjectively) on HR measurement error from photoplethysmographic devices (Sañudo et al. 2019; Etiwy et al. 2019; Bent et al. 2020; Ray et al. 2021). All four studies measured HR during rest, stationary cycling, and/or treadmill walking, which are activities with little to no motion artifact and that have been previously shown to produce better photoplethysmographic HR accuracy (Spierer et al. 2015; Pasadyn et al. 2019) compared to modes of exercise with greater motion artifact like running (Hermand et al. 2019). Similar to others, we also observed mean differences of 1–6 bpm between the wearable device and criterion HR measures (Sañudo et al. 2019) across a range of intensities.

Comparing the current study with studies that have concluded skin color negatively affects photoplethysmographic performance is challenging because of methodological differences and lack of data reporting. For example, three studies did not measure HR (Fallow et al. 2013; Hochstadt et al. 2020; Puranen et al. 2020), one study used another photoplethysmographic device for the criterion HR measurement (Preejith et al. 2016), one study did not report the data to support their conclusion of greater error in HR measurement at darker skin tones (Shcherbina et al. 2017), and another determined that skin color was correlated with measurement bias but the magnitude was negligible, although the exact magnitude was not reported (Hermand et al. 2019). Given the limited data available to draw comparisons, our results for the SlateSafety device are in line with Hermand et al. (2019), who also tested a photoplethysmographic device worn around the upper arm, and Hung et al. (2025), who tested a wrist-worn Fitbit. However, given that different brands, sensor designs, and algorithms were used across studies, it is difficult to identify why these devices produced significant results when others did not. To our knowledge, the present study is also the first to report the rates of missing and outlier data by skin color. As such, we are unable to determine how these findings compare to prior studies.

Additionally, all of the aforementioned studies investigating the relationship between skin tone and photoplethysmographic HR used a subjective, categorical rating of skin color: Fitzpatrick Skin Type (Fallow et al. 2013; Shcherbina et al. 2017; Sañudo et al. 2019; Hermand et al. 2019; Bent et al. 2020; Hochstadt et al. 2020; Puranen et al. 2020; Ray et al. 2021; Hung et al. 2025), “white, black, other” (Etiwy et al. 2019), or “fair, moderate, dark” (Preejith et al. 2016). As previously noted, using a subjective assessment of skin pigmentation has been shown to have poor reliability and validity (Pershing et al. 2008; Branigan et al. 2013). Several of these studies are further limited by unequal distribution of skin tones (Preejith et al. 2016; Etiwy et al. 2019; Hochstadt et al. 2020; Ray et al. 2021) and/or a narrow range in skin tones on the Fitzpatrick scale (Sañudo et al. 2019). Nonetheless, our findings are consistent with others who examined the influence of skin tone—albeit measured subjectively—on HR measurement error (Sañudo et al. 2019; Etiwy et al. 2019; Hermand et al. 2019; Bent et al. 2020; Ray et al. 2021; Hung et al. 2025). This study expands the current body of knowledge by showing that skin pigmentation, when measured objectively using ITA°, does not appear to influence the accuracy of photoplethysmography-derived HR measurement for the Apple Watch Series 8 or the Garmin vivosmart 5, but may have limited impact on the accuracy of arm-band style devices like the SlateSafety BAND when used in a healthy, relatively young, non-obese sample during stationary cycling.

Two other studies have quantified skin pigmentation as ITA° but did not explore the accuracy of HR measured by photoplethysmographic devices. Instead, they examined the relationship between bias in oxygen saturation measurement using infrared light and ITA°. Fawzy et al. (2024) observed greater bias in individuals with darkly pigmented skin when defined as ITA° less than − 30°, while Guo et al. (2023) found that correcting for ITA° improved oxygen saturation measurements from a novel wrist-worn device. Pulse oximeters, a type of photoplethysmographic device, measure the amount of light that is reflected vs. absorbed by the vasculature over time and predict oxygen saturation from standard curves (Chan et al. 2013). Given that melanin and hemoglobin both have low absorption properties for red and infrared light (wavelengths > 625 nm), and oxygenated blood scatters more light than deoxygenated blood (Sardar et al. 2001), higher melanin content would be expected to artificially inflate oxygen saturation measures. Indeed, Fawzy et al. (2024) observed a 1% overestimation of oxygen saturation in individuals with darker skin. In contrast to pulse oximetry, HR detection from a photoplethysmographic signal uses a peak detection algorithm (often with filtering, to account for motion artifact and other noise), which relies on the shape of the photoplethysmographic wave rather than the absolute amplitude (Mejía-Mejía et al. 2022). Considering the findings involving pulse oximetry, we speculate the wearable devices tested in our study were able to detect the pulsatile nature of blood flow and thereby accurately estimate HR, despite a reduction in light transmission in the presence of greater melanin content. Moreover, our use of stationary cycling likely resulted in little motion artifact, thereby isolating the impact of epidermal melanin on photoplethysmographic HR accuracy.

While we found no apparent influence of skin pigmentation on photoplethysmographic HR measurement accuracy for the Apple and Garmin devices, and a minimal impact (~ 1 bpm) for the SlateSafety BAND V2, these findings should be applied cautiously. It has been repeatedly documented in the literature that melanin reduces light transmission and photoplethysmographic signal quality (Zonios et al. 2001; Fallow et al. 2013; Hochstadt et al. 2020; Puranen et al. 2020), and that darker skin results in greater error in HR measurement during activities with relatively greater upper limb movement (Shcherbina et al. 2017; Hermand et al. 2019). It is also important to note that the unexplained missing data and outliers both occurred more frequently in our participants with dark skin (ITA° < 10°) across all devices, which may have contributed to null findings in our assessment of the influence of ITA° on MAE_HR_. In addition, it is important to note that the overall rate of data loss for unidentifiable reasons from the SlateSafety (20%) device was substantial, and an end-user may be unaware of this unless they examined the raw data themselves.

One limitation of this study is that we were unable to determine whether the participants with darker skin and higher epidermal melanin content experienced a reduced photoplethysmographic signal as we do not have access to the raw photoplethysmographic data or the algorithms used by the devices we tested. Regardless, the accuracy of available photoplethysmographic HR measurement was generally unaffected. We feel confident in this conclusion as we carefully considered the sample size needed to detect significant differences, and were able to obtain a diverse sample (46% self-reported persons of color and 36% of all participants had ITA° < 10°). We cannot rule out the possibility that introducing additional common sources of error, like motion artifact, may disproportionately limit the ability of these devices’ algorithms to accurately measure HR in individuals with darker skin outside of a highly controlled laboratory setting. That said, we examined three commercially available devices across rest, recovery, and very light to vigorous exercise intensities, which showed no impact of skin pigmentation on device accuracy (Apple and Garmin watches) and the difference in accuracy observed for the SlateSafety device was of limited clinical significance (~ 1 bpm). Further investigation is required to determine whether there is an interaction between epidermal melanin and limb movement, especially during non-cycling exercise.

To our knowledge, this is the first study to assess the impact of objectively measured skin pigmentation on photoplethysmographic HR accuracy. We provide evidence that, under isolated conditions, darker skin does not appear to reduce the accuracy of wrist-worn HR measured by the tested photoplethysmographic devices during rest, a range of exercise intensities while stationary cycling, or seated recovery. However, our data provide evidence that skin pigmentation negatively affects data quality, as missing and erroneous data occurred at a disproportionately higher rate in participants with darker skin. Of the wearable devices included in this study, the wrist-worn devices (Apple and Garmin watches) demonstrated excellent agreement with criterion HR, and the arm-band device (SlateSafety BAND) produced good agreement.

## Supplementary Information

Below is the link to the electronic supplementary material.

Supplementary file1 (PDF 141 kb)

Supplementary file2 (PDF 37 kb)

Supplementary file3 (PDF 131 kb)

## Data Availability

Data are available from the corresponding author (AMM) upon reasonable request.
