# Supplementary material for: Influence of skin pigmentation on the accuracy and data quality of photoplethysmographic heart rate measurement during exercise
Source: Eur J Appl Physiol. 2025 Sep 18;126(2):1057–66. doi: 10.1007/s00421-025-05977-x (PMC12948776; doi:10.1007/s00421-025-05977-x)
Supplement: Supplementary file 1 — Supplementary file1 (PDF 141 kb) [file 421_2025_5977_MOESM1_ESM.pdf]

Title: Influence of Skin Pigmentation on the Accuracy of Photoplethysmographic Heart Rate Measurement During Exercise

Journal: European Journal of Applied Physiology

Authors: Anne M. Mulholland[1,2], Hayley V. MacDonald[2], Elroy J. Aguiar[2], Jonathan E. Wingo[2]
[1]Department of Exercise Science, Mercer University, Macon, GA, USA
[2]Department of Kinesiology, The University of Alabama, Tuscaloosa, AL, USA

Corresponding author: Anne M. Mulholland

Email: mulholland_a@mercer.edu

Mean absolute percent error data for the 3 tested devices: Apple Watch Series 8, Garmin vivosmart 5, SlateSafety BAND V2.

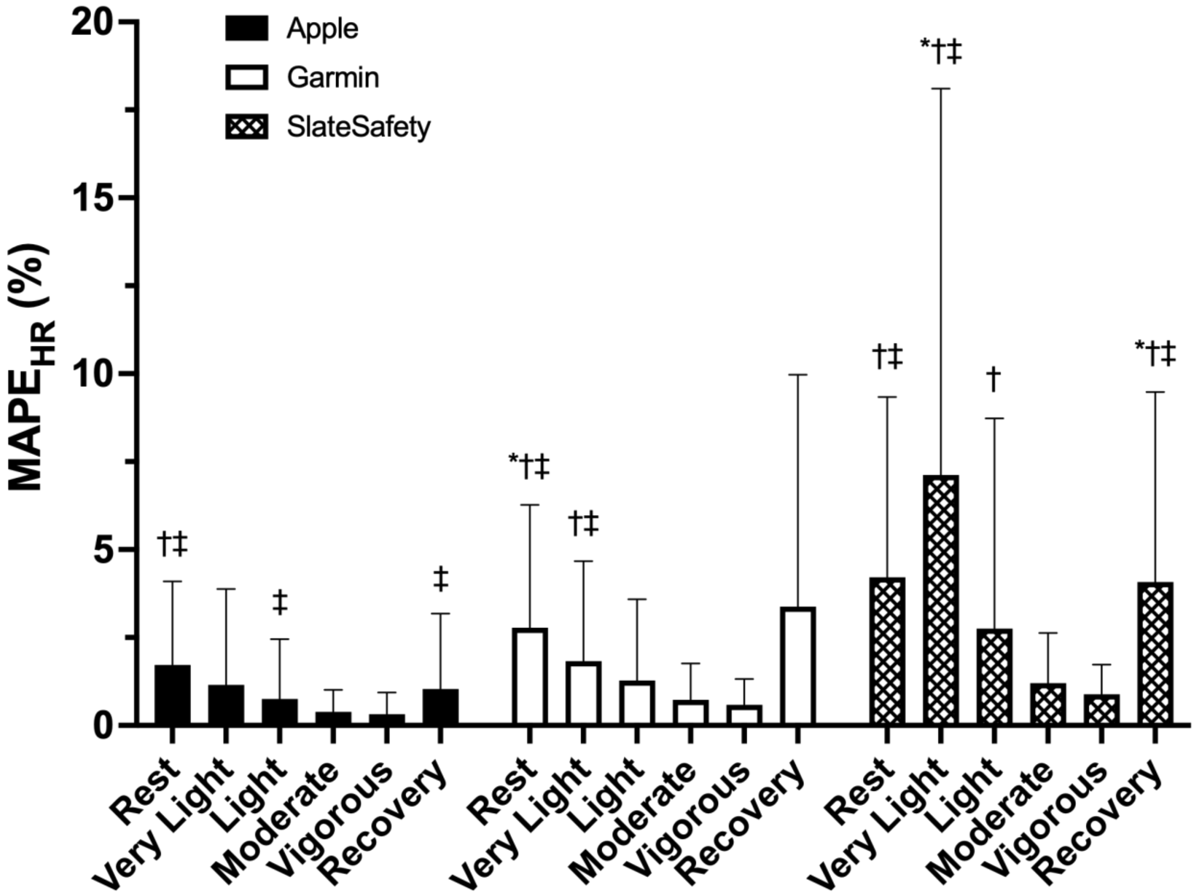

Fig. OR1 Mean ± SD mean absolute percent error of heart rate measurement ($MAPE_{HR}$) between the criterion device (Polar H10) and each photoplethysmographic device during rest, exercise (very light, light, moderate, and vigorous intensities), and recovery. * $P<0.05$ compared to light intensity within the same device; † $P<0.05$ compared to moderate intensity within the same device; ‡ $P<0.05$ compared to vigorous intensity within the same device
