# Supplementary material for: Influence of skin pigmentation on the accuracy and data quality of photoplethysmographic heart rate measurement during exercise
Source: Eur J Appl Physiol. 2025 Sep 18;126(2):1057–66. doi: 10.1007/s00421-025-05977-x (PMC12948776; doi:10.1007/s00421-025-05977-x)
Supplement: Supplementary file 2 — Supplementary file2 (PDF 37 kb) [file 421_2025_5977_MOESM2_ESM.pdf]

Title: Influence of Skin Pigmentation on the Accuracy of Photoplethysmographic Heart Rate Measurement During Exercise

Journal: European Journal of Applied Physiology

Authors: Anne M. Mulholland[1,2], Hayley V. MacDonald[2], Elroy J. Aguiar[2], Jonathan E. Wingo[2]
[1]Department of Exercise Science, Mercer University, Macon, GA, USA
[2]Department of Kinesiology, The University of Alabama, Tuscaloosa, AL, USA

Corresponding author: Anne M. Mulholland

Email: mulholland_a@mercer.edu

Additional data quality information for the 3 tested devices: Apple Watch Series 8, Garmin vivosmart 5, and SlateSafety BAND V2. Missing data counts are given in Table OR1 and outlier data counts are given in OR2.

Table OR1. Missing data, counted as 30-s epochs, by intensity for each device.

Intensity	Apple Watch Series 8	Garmin vivosmart 5	SlateSafety BAND V2
Rest	30	39	47
Very Light	3	1	136
Light	3	1	72
Moderate	1	1	62
Vigorous	8	9	99
Recovery	9	1	101
Total missing data	54	52*	517

*Note: Garmin data for 2 participants was not able to be downloaded from the device after recording the session. Those data are not included in this table.

Table OR2. Outlier occurrence by intensity for each device.

Intensity	Apple Watch Series 8	Garmin vivosmart 5	SlateSafety BAND V2
Rest	2	2	0
Very Light	16	8	51
Light	4	5	9
Moderate	1	1	0
Vigorous	2	1	0
Recovery	6	28	9
Total outliers	31	45	69
