# Supplementary material for: Influence of skin pigmentation on the accuracy and data quality of photoplethysmographic heart rate measurement during exercise
Source: Eur J Appl Physiol. 2025 Sep 18;126(2):1057–66. doi: 10.1007/s00421-025-05977-x (PMC12948776; doi:10.1007/s00421-025-05977-x)
Supplement: Supplementary file 3 — Supplementary file3 (PDF 131 kb) [file 421_2025_5977_MOESM3_ESM.pdf]

Title: Influence of Skin Pigmentation on the Accuracy of Photoplethysmographic Heart Rate Measurement During Exercise

Journal: European Journal of Applied Physiology

Authors: Anne M. Mulholland[1,2], Hayley V. MacDonald[2], Elroy J. Aguiar[2], Jonathan E. Wingo[2]
[1]Department of Exercise Science, Mercer University, Macon, GA, USA
[2]Department of Kinesiology, The University of Alabama, Tuscaloosa, AL, USA

Corresponding author: Anne M. Mulholland

Email: mulholland_a@mercer.edu

Each table contains full model building results from the linear mixed-effects analysis performed for each of the tested devices: Apple Watch Series 8 (Table OR3), Garmin vivosmart 5 (Table OR4), and SlateSafety BAND V2 (Table OR5).

Table OR3. Prediction of mean absolute error in heart rate measurement by the Apple Watch Series 8.

		Coefficients		Likelihood ratio test[a]		
Model	*Constant*	*ITA°*	*Criterion HR*	X^2	*Comparison model*	*Marginal* R^2
0	0.694*					
1	0.701*	−0.001		0.253	0	<0.001
2	1.433*		−0.006*	102.07*	0	0.034

[a] all chi-square tests used 1 degree of freedom
ITA°, individual typology angle; HR, heart rate
* P<0.05

Table OR4. Prediction of mean absolute error in heart rate measurement by the Garmin vivosmart 5.

		Coefficients		Likelihood ratio test[a]		
Model	*Constant*	*ITA°*	*Criterion HR*	X^2	*Comparison model*	*Marginal* R^2
0	1.324*					
1	1.279*	0.003		1.145	0	0.003
2	2.779*		−0.013*	106.28*	0	0.040

[a] all chi-square tests used 1 degree of freedom
ITA°, individual typology angle; HR, heart rate
* P<0.05

Table OR5. Prediction of mean absolute error in heart rate measurement by the SlateSafety BAND V2.

		Coefficients		Likelihood ratio test[a]		
Model	*Constant*	*ITA°*	*Criterion HR*	X^2	*Comparison model*	*Marginal* R^2
0	2.434*					
1	2.599*	−0.011*		10.446*	0	0.019
2	5.160*	−0.011*	−0.022*	103.200*	1	0.058

[a] all chi-square tests used 1 degree of freedom
ITA°, individual typology angle; HR, heart rate
* P<0.05
